# Biosynthesis of heme O in intraerythrocytic stages of *Plasmodium falciparum* and potential inhibitors of this pathway

**DOI:** 10.1038/s41598-019-55506-y

**Published:** 2019-12-17

**Authors:** Raquel M. Simão-Gurge, Gerhard Wunderlich, Julia A. Cricco, Eliana F. Galindo Cubillos, Antonio Doménech-Carbó, Gerardo Cebrián-Torrejón, Fernando G. Almeida, Brenda A. Cirulli, Alejandro M. Katzin

**Affiliations:** 1 Department of Parasitology, Institute of Biomedical Sciences, University of São Paulo, São Paulo, Brazil; 2 Instituto de Biología Molecular y Celular de Rosario (IBR), Consejo Nacional de Investigaciones Científicas y Técnicas CONICET – Facultad de Ciencias Bioquímicas y Farmacéuticas, Universidad Nacional de Rosario, Rosario, Argentina; 3 Department of Analytical Chemistry, Faculty of Chemistry, University of Valencia, Valencia, Spain; 4 Laboratoire COVACHIM-M2E EA 3592, Université des Antilles, 97157 Pointe-à-Pitre Cedex, Guadeloupe France

**Keywords:** Biophysical chemistry, Parasite physiology

## Abstract

A number of antimalarial drugs interfere with the electron transport chain and heme-related reactions; however, the biosynthesis of heme derivatives in *Plasmodium* parasites has not been fully elucidated. Here, we characterized the steps that lead to the farnesylation of heme. After the identification of a gene encoding heme O synthase, we identified heme O synthesis in blood stage parasites through the incorporation of radioactive precursors. The presence of heme O synthesis in intraerythrocytic stages of *Plasmodium falciparum* was confirmed by mass spectrometry. Inabenfide and uniconazole–P appeared to interfere in heme synthesis, accordingly, parasite growth was also affected by the addition of these drugs. We conclude that heme O synthesis occurs in blood stage-*P. falciparum* and this pathway could be a potential target for antimalarial drugs.

## Introduction

Malaria is one of the most important tropical diseases, and the World Health Organization regards malaria as one of the top ten causes of death worldwide. Approximately 200 million clinical cases occur every year, causing the death of approximately 430 thousand persons^1^. Currently, the main option to treat the disease is treatment with artemisinin-based compounds (ACTs), which contain artemisinin in combination with other drugs such as lumefantrine or atovaquone; these compounds are currently the most effective drugs to treat malaria^2^.

Malaria is caused by protozoan parasites of the genus *Plasmodium*, including *P. falciparum*, *P. vivax*, *P. ovale*, *P. malariae* and *P. knowlesi*, in order of importance. *P. falciparum* is responsible for the majority of malaria deaths globally, and it is the most prevalent species in sub-Saharan Africa.

There is a rapid emergence of drug resistance in *Plasmodium* spp. to existing antimalarial drugs and this has motivated the search for novel targets as well as derivatives from original molecules with improved activity against validated drug targets.

One target for the evaluation of potential antimalarial compounds is the isoprenoid synthesis, which occurs via the 2-C-methyl-D-erythritol-4-phosphate (MEP) pathway in *P. falciparum*. It is also present in some plants and most bacteria^3–7^. In contrast, most animal cells, several eubacteria, archaea, and fungi synthesize isoprenoid precursors through the mevalonate pathway^8,9^. In *P. falciparum*, the biosynthesis of the first intermediates of the isoprenoid pathway occurs in the organelle known as apicoplast, a vestigial plastid present in several protozoan parasites of the subphylum Apicomplexa^10^. Since heme O derives from the isoprenoid pathway, it may be an important target for drugs different from quinoline compounds.

Another target of many antimalarials is heme B, or heme, a product released during hemoglobin degradation. *P. falciparum* has developed a mechanism to defend itself against the accumulation of heme B by polymerizing the porphyrin ring to crystalline hemozoin. Quinoline drugs inhibit this polymerization by forming a heme-drug complex. This causes the accumulation of heme B, which is then toxic to *Plasmodium spp*.^11,12^. Intriguingly, the parasite possesses its own synthesis for heme B^13^ which suggests that heme B is an important cofactor for the parasite. In order to use heme B in the electron transport chain, an intermediate of the isoprenoid pathway - farnesyl-pyrophosphate (FPP) - is covalently bound to heme B resulting in heme O. This reaction is catalyzed by the heme O synthase (HOS). Heme O is an essential molecule for several living organisms, as it is the first intermediate in heme A synthesis. Both heme O and heme A are critical components of the mitochondrial electron transport chain: heme O is a cofactor for terminal oxidases in *E. coli*, and heme A acts as a cofactor of several bacterial and all eukaryotic cytochrome c oxidases^14^, catalyzing the reduction of O_2_ to H_2_O^14,15^.

Previous studies have demonstrated that antifungal azoles, such as ketoconazole (Keto), myconazole (Myco), and clotrimazole (CLT), effectively inhibit the growth of *P. falciparum in vitro*, and the mechanism involved is related to the inhibition of heme polymerization and the degradation of reduced glutathione-dependent heme^16^. Another study showed that two other antifungals, inabenfide (INA) and uniconazole–P (UNP), inhibit the growth in both apicomplexan parasites *P. falciparum* and *Toxoplasma gondii*^17^. These drugs act by decreasing the synthesis of isoprenic products in plants; however, in *P. falciparum*, the mode of action of these drugs is unknown.

The aim of this work was to identify an active biosynthesis pathway of heme O as intermediary of heme A as well as to analyze the effect of potential inhibitors.

## Results and Discussion

Reports of looming resistance to artemisinin and its derivatives are a major concern for malarial treatment^18,19^, and the fight against artemisinin non-responsive parasites, the elucidation of exploitable drug targets and the development of new drugs are becoming urgent issues. Recently, a thorough analysis of druggable targets in *Plasmodium* was conducted and emerging resistance markers were characterized^20^.

We have been focusing on the biosynthesis of derivatives of the isoprenoid pathway in *P. falciparum*^21^. Despite the fact that the intraerythrocytic parasite lives in an environment that is particularly rich in heme, it has retained its own heme synthesis pathway. However, unlike heme B, the isoprenoid-linked heme O and heme A cannot be obtained from the host. Given that they are essential in other organisms, the characterization of heme farnesylation may reveal important targets against *Plasmodium*^22^.

### Identification of a gene encoding heme O synthase

For the characterization of heme O biosynthesis, we identified a gene encoding a protein with sequence homology to heme O synthase (HOS) in the genome of *P. falciparum*^23^, an enzyme required for farnesylation of heme.

In *Saccharomyces cerevisiae*, HOS, also referred to as COX10^24^, is a mitochondrial inner membrane protein that forms a multimeric complex of ~300 kDa. Subsequently, heme A synthase (HAS) oxidizes heme O to yield heme A, a cofactor of cytochrome *c* oxidase (COX) or complex IV of the mitochondrial respiratory chain. COX has several subunits, three of which are encoded in mitochondrial DNA; these are referred to as COX1, COX2 and COX3. The stability of the COX10 oligomer seems to depend on the presence of freshly synthesized COX1 and its intermediates^25^.

The sequence identified in the *P. falciparum* genome that encodes a putative COX10, PF3D7_0519300, shares more than 60% amino acid similarity to previously characterized enzymes from other organisms. Furthermore, the residues considered relevant for the catalytic activity of COX10 were conserved in the *P. falciparum* sequence (Supplementary Information, Fig. S2); these are N196, R212, R216 and H317 following *S. cerevisiae* COX10 numbering^26,27^. The sequence was scanned for potential transmembrane regions, and five were identified in PF3D7_0519300, similar to other COX10 proteins (Supplementary Information Fig. S2). A phylogenetic tree (Supplementary Information Fig. S3) showing the evolutionary relationship among different COX10 sequences revealed a close relationship between the *P. falciparum* and *S. cerevisiae* enzymes. The enzyme COX10 from *S. cerevisiae* had been characterized^28^. These data suggest that PF3D7_0519300 in fact encodes the *P. falciparum* version of COX10.

In addition, through the phylogenetic tree of COX10 (Supplementary Information Fig. S3), the similarity of *Plasmodium* spp. COX10 with the enzyme from other organisms of the apicomplexan phylum was compared. Within the genus of *Plasmodium*, *P. falciparum* COX10 is closest to *P. reichenowi* COX10, what is expected given the similarities in most genes between these species^29^. First, we focused on the characterization of heme O because not all organisms biosynthesize heme A^14^.

### Subcellular location of COX10

Since the data suggest that PF3D7_0519300 encodes COX10 in *P. falciparum*, we considered it important to confirm its subcellular location. Using GFP-tagged COX10, we conducted fluorescence microscopy experiments using MitoRed and DAPI staining as controls (Supplementary Information Fig. S4).

Since the apicoplast and the mitochondrion are close, it is mandatory to use specific markers to clearly differentiate them. Therefore, to identify if the GFP fluorescence signal from the Cox10-GFP fusion originated from either the mitochondrion or the apicoplast, or both, we used a mitochondrial marker and an apicoplast-specific antibody produced according to Tonkin *et al*.^30^ (produced by FastBio, Ribeirão Preto, Brazil). With this, we verified that COX10 signal (as green fluorescence) is mostly overlapping with the mitochondrial red signal and, to a smaller extent, in the nucleus. Besides that, DAPI also stains mitochondrial DNA, which may explain a partial overlap between the DAPI and the GFP signal. In contrast, the overlapping fluorescence observed within the apicoplast is not fully overlapping with the signal of Cox10-GFP, suggesting that PfCOX10 is probably not located in the apicoplast (Supplementary Information Fig. S4). Based on the COX10 sequence obtained from PlasmoDB, there is also no predicted signal sequence that may direct the protein to the Apicoplast (see entry for PF3D7_0519300 in www.plasmoDB.org).

In most eukaryotes, including humans and *S. cerevisiae*, cytochrome c oxidase (COX) is the terminal component of the mitochondrial respiratory chain. Intriguingly, neither plasmoDB.org nor the conserved domain server at NCBI predicted a mitochondrial localization signal.

A nuclear gene encodes human and *S. cerevisae* COX10, which is not a structural subunit but is required for heme A synthesis^31^. The human or yeast COX10 enzyme is located in the mitochondrion and is necessary for the synthesis of COX^28^. The localization of the putative plasmodial COX10 in the mitochondrion suggests that the *P. falciparum* cox10 gene indeed encodes the plasmodial COX10 enzyme.

### Biosynthesis of heme O

**Figure 1:**
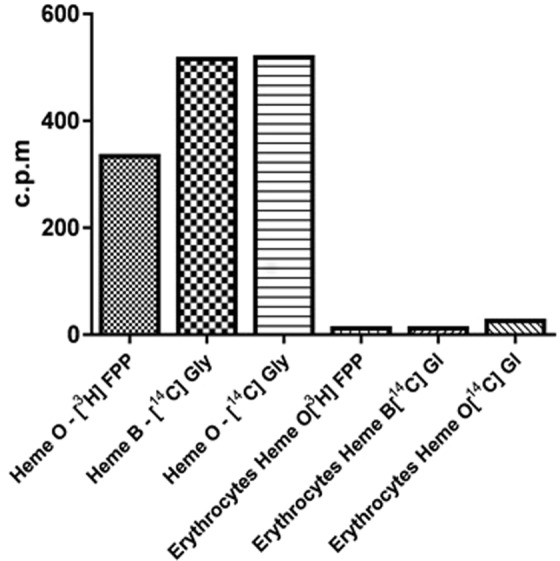
*P. falciparum* synthesizes heme O. Parasitized erythrocytes and non-parasitized erythrocytes were labeled with [1-(n)-^3^H]-FPP or with [U-^14^C]-glycine, each extract was purified by affinity columns and the peaks were analyzed by a scintillator. The fraction eluted with 80% ACN, which elutes heme B, presents the radioactive incorporation of glycine and the fraction eluted with DMSO, contained radioactive heme O. Heme O-[^3^H]FPP is the extract of parasitized erythrocytes labeled with [1-(n)-^3^H]-FPP and eluted with DMSO; Heme B-[^14^C]Gly is the extract of parasitized erythrocytes labeled with [U-^14^C]-glycine eluted with 80% ACN; Heme O-[^14^C]Gly is the extract of parasitized erythrocytes labeled with [U-^14^C]-glycine eluted with DMSO; Erythrocytes Heme O-[^3^H]FPP is the extract erythrocytes labeled with [1-(n)-^3^H]-FPP and eluted with DMSO; Erythrocytes Heme B-[^14^C]Gl is the extract of erythrocytes labeled with [U-^14^C]-glycine and eluted with 80% ACN; Erythrocytes Heme O-[^14^C]Gl is the extract of erythrocytes labeled with [U-^14^C]-glycine and eluted with DMSO.

We first characterized heme O using metabolic labeling with [1-(n)-^3^H]-FPP (direct precursor for the formation of heme O) or [U-^14^C]-glycine (the initial precursor of the heme pathway). The detection of radiolabeled heme O and heme B from schizonts showed that there is an active synthesis of heme B and heme O (Fig. 1) which is absent in non-parasitized erythrocytes. As heme B biosynthesis has already been described, we used these data as a positive control for the experiment^32,33^. The identification of standard of heme B is shown in Supplementary Information Fig. S5, and based on data published by Brown *et al*.^32,33^.

**Figure 2:**
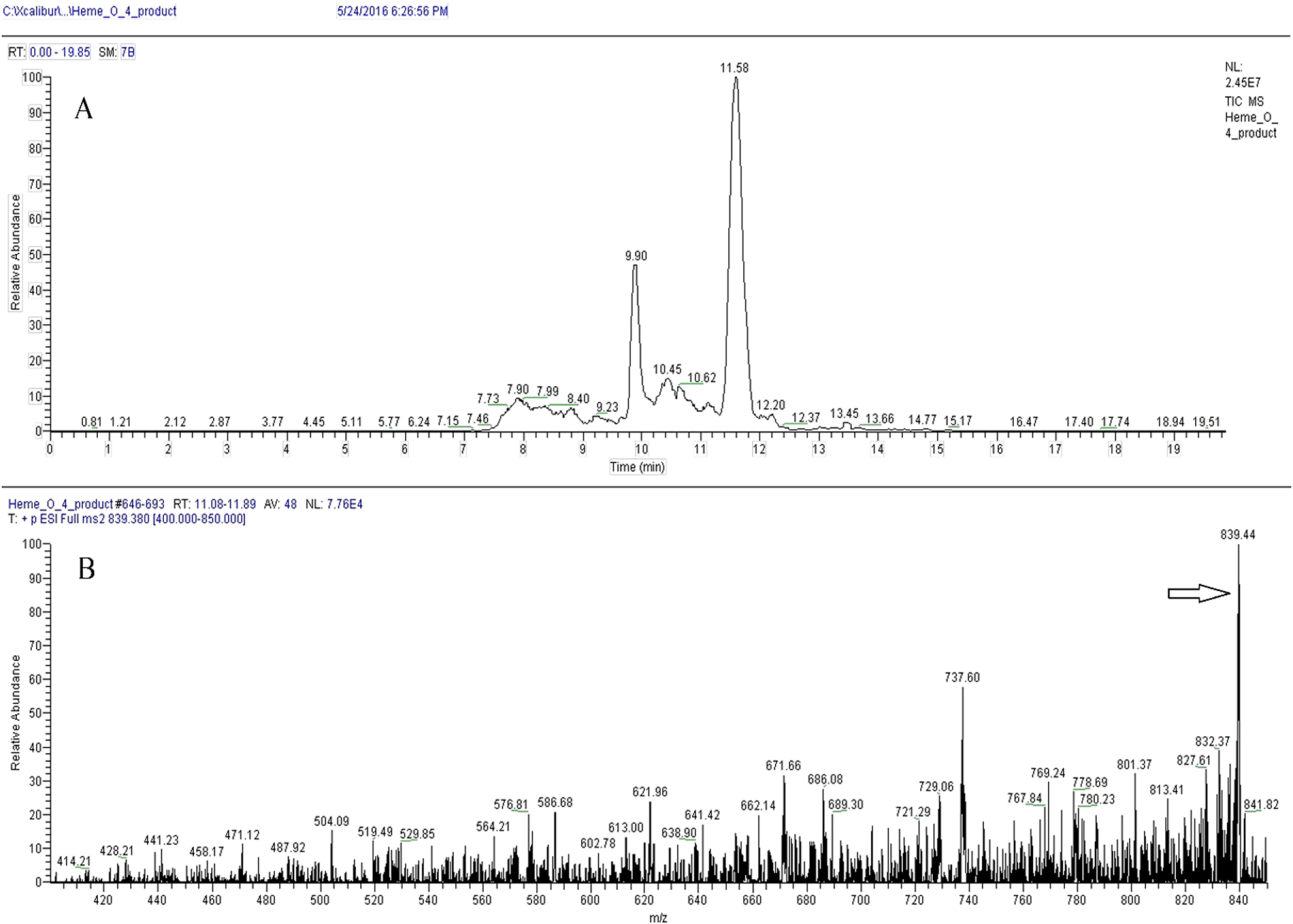
Identification of heme O by LC-MS/MS in schizonts extracts. Heme O was separated using C18 Vac columns and eluted with DMSO and analyzed by LC-MS / MS. (**A**) Heme O separation was performed using a Hypersil Gold C18 Column (see material and methods: Mass Spectrometry; LC-MS/MS) and the peak with retention time of 11.58 was analyzed by MS/MS. (**B)** MS/MS of peak of 11.58 showing the m/z of heme O (arrow, 839.4, compatible with its calculated mass).

**Figure 3:**
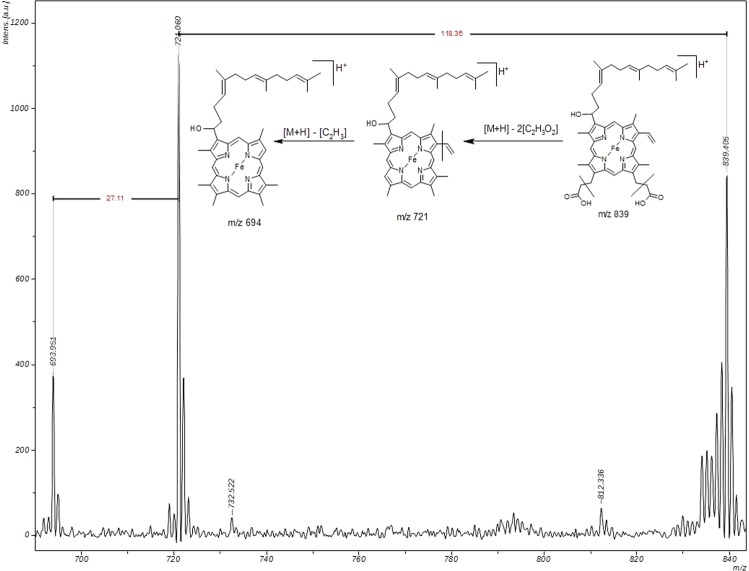
Identification of heme O by MALDI-TOF/TOF in schizonts extracts. Heme O was separated using C18 Vac columns eluted with DMSO, and analyzed in MALDI-TOF/TOF. Heme O represented by the parent mass m/z 839.405 (compatible with its calculated mass), followed by its fragments with masses m/z 721.060 and m/z 693.951.

To confirm the presence of heme O in unlabeled parasites, two different analyses were performed using mass spectrometry (Figs. 2 and 3). In a first step, the parasite extract was loaded on Sep-Pak C18 columns and the peak corresponding to heme O was analyzed by LC-MS/MS and MALDI-TOF/TOF. For this purpose, a LC-MS/MS method was developed, as described in the methods section, with the instrument set to assess the product ions for the compound, including m/z 839, as shown in Fig. 2B. The presented spectrum was obtained from the peak at the retention time point 11.58 minutes (Fig. 2A) and shows a product of m/z 839, and the two most important ion products are m/z 737 and m/z 671 (Fig. 2B), coincident with heme O.

Complementarily, a MALDI-TOF/TOF analysis was carried out, and the results are presented in Fig. 3. Since the fragmentation pattern is different in the used instruments as previously described for peptides^34^, two different ion products could be visualized for the same parent ion m/z 839 of heme O.

Accordingly, Fig. 3 shows the spectrum of heme O, represented by the parent mass m/z 839.405, followed by its fragments with masses m/z 721.060 and m/z 693.951. The peak m/z 721.060 is a result of the neutral loss of two molecules of [C_2_H_3_O_2_] = 118.35 Da from the peak m/z 839.040. The peak m/z 693.951 is a result of the subsequent loss of a molecule [C_2_H_3_] = 27.11 Da from the peak m/z 721.060. Additionally, the experimental isotope distribution of m/z 839.040 corroborates with the theoretical isotope distribution and relative intensities of m/z 837.387 (6.3), m/z 838.390 (3.5), m/z 839.383 (100.0), m/z 840.386 (57.3), m/z 841.389 (17.5), m/z 842.393 (3.7), and m/z 843.396 (0.6) for a protonated compound with a molecular formula of C_49_H_58_O_5_N_4_Fe. These data confirm that the identified compound isolated from the parasite extract at a retention time of 11.58 minutes is heme O.

Again, it is important to mention that two different ionization methods - electrospray (ESI) for LC-MS/MS and MALDI - were applied and both techniques provided results compatible with the molecular ion and fragment pattern of heme O.

As a control, the acquired spectra from the extracts of transformed bacteria expressing the *Bacillus subtilis* CtaB protein, which corresponds to heme O synthase, and the spectra from the parasite extracts were compared. In both, we observed the ion m/z 617 corresponding to heme B (Supplementary Information Figs. S5–S7). This underscores that mass-spectrometric analysis is a reliable methodology.

According to Ke *et al*.^35^, the heme biosynthetic pathway is not essential for asexual parasites stage. The reason why this pathway is still active in the parasite during the asexual stage is intriguing because free heme may be toxic to the parasite. However, the level of endogenously synthesized heme in parasites is much lower than that produced via hemoglobin digestion. It is possible that the parasite keeps this pathway active to supply heme for heme O biosynthesis, since farnesylated heme is essential for the biosynthesis of heme A (with heme O as intermediate), cytochrome *c*, or to supply heme for other heme-proteins. This occurs probably due to the changing availability of host-derived heme, which profoundly differs in dependence on the host at every stage. While during liver stage heme is probably scarce, an excess is present in erythrocytes. In a recent study on the role of dual heme sources in malaria parasite growth and development, the first enzyme, δ-aminolevulinate synthase (ALAS), and the last enzyme, ferrochelatase (FC), in the heme pathway of *Plasmodium berghei* (Pb) had been knocked out. The wild-type and knockout (KO) parasites had similar intraerythrocytic growth patterns in mice. Upon *in vitro* radiolabeling of heme in Pb-infected mouse reticulocytes and *P. falciparum*-infected human RBCs using [4-^14^C]-aminolevulinic acid (ALA), parasites were found to incorporate in both hosts hemoglobin-heme and parasite-synthesized heme into hemozoin and mitochondrial cytochromes. The similar fates of the two heme sources suggest that parasites may obtain heme from different origins, emphasizing the biological importance of heme availability in the parasite^13^. Notably, during a genome wide piggyback-mediated insertion-mutation approach, the COX10 gene was deemed important for survival in blood stage parasites^36^.

Having shown the existence of heme O synthesis, we set out to determine whether the drugs INA and UNP specifically interfere with heme B and/or heme O synthesis. Both drugs perturb organelle structure and food vacuole morphology in schizonts^17,37^. In plants, INA and UNP inhibit the ent-kaurene oxidase (KO), a member of the cytochrome P450 monooxygenase family (CYP) that catalyzes three successive oxidations of the 4-methyl group of ent-kaurene, resulting in kaurenoic acid, a key intermediate in gibberellin (GA) biosynthesis^38^.

Since a number of plant-exclusive isoprenic metabolites have also been found in *P. falciparum*, we investigated the possible route of gibberellin biosynthesis. However, we were unable to find hints of such pathway. The results obtained by metabolic labeling and recovery tests, where cultures were treated with INA or UNP and enriched with the theoretically inhibited end product (in this case, GA4) were negative (data not shown). Knowing that other antifungal agents such as azole act in the heme pathway^16^, we decided to investigate whether INA and UNP had their mechanism of action related to the heme biosynthesis or heme farnesylation pathway.

### INA and UNP as potential inhibitors of heme O biosynthesis

**Figure 4:**
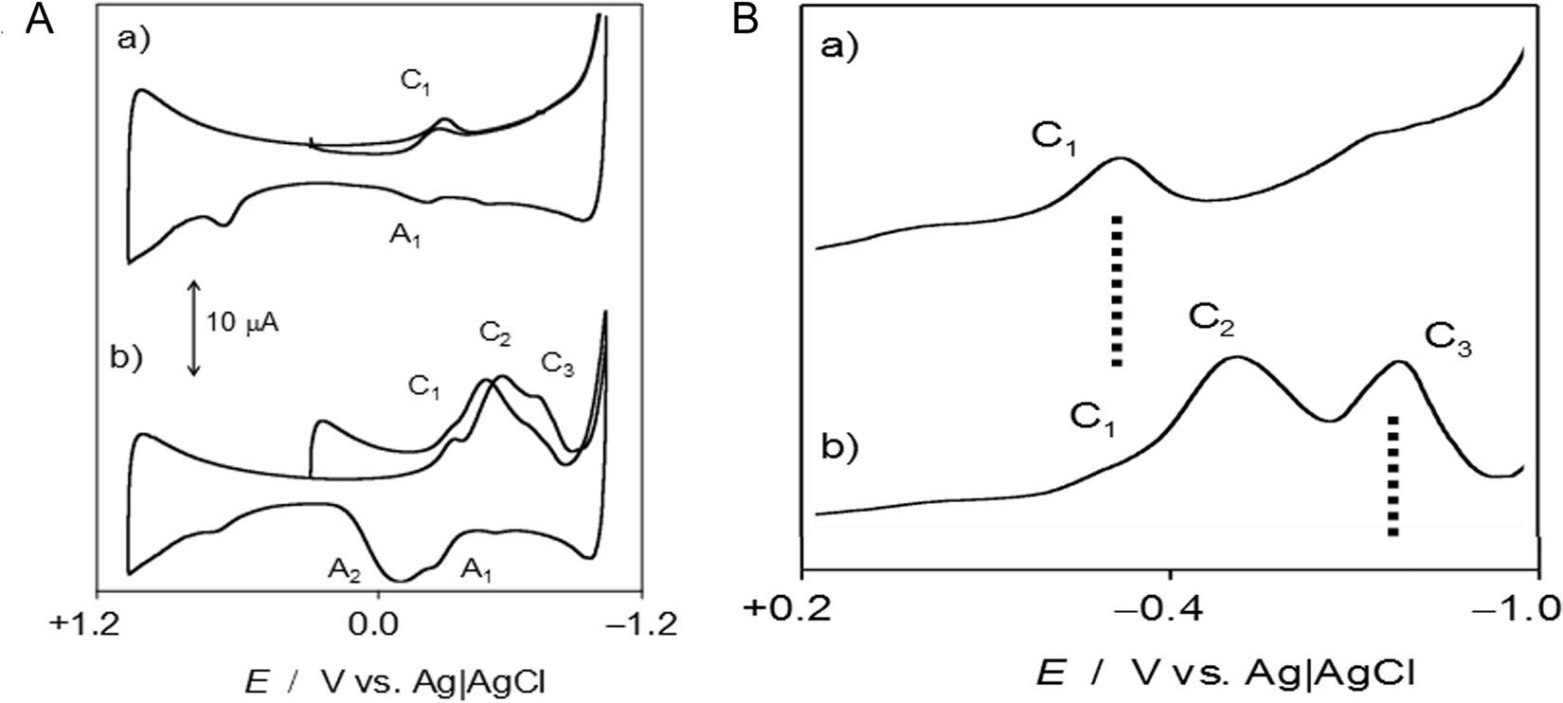
(**A**) The cyclic voltammograms of glass carbon electrodes modified with (a) uninfected erythrocytes and (b) *P. falciparum*-infected erythrocytes in contact with 0.1 M PBS at pH 7.4. The potential scan rate was 50 mV/s. Semi-derivative convolution was performed to increase signal resolution. (**B**) The square wave voltammograms of glassy carbon electrodes modified with (a) uninfected erythrocytes, (b) *P. falciparum*-infected erythrocytes. The potential scan was initiated at +0.2 V in the negative direction; the potential step increment was 4 mV. The square wave amplitude was 25 mV at a frequency of 5 Hz.

First, we investigated whether INA and UNP inhibited heme B or heme O biosynthesis. We conducted an electrochemical oxidation/reduction test to determine if these drugs interfered in any way with heme. To test the differences in the redox behavior between healthy and *Plasmodium*-infected erythrocytes, films of both specimens were prepared on glass carbon electrodes and immersed in PBS at pH 7.2 (Fig. 4A). The voltammetry response of the uninfected erythrocyte sample was a pair of signals at −0.34 (cathodic, C_1_) and −0.26 V (anodic, A_1_) against Ag/AgCl, which can be attributed to the Fe(III)-heme/Fe(II)-heme one-electron interconversion. The symmetry of the peak profiles and their relatively low separation in the potential scale indicates that the electrochemical process possesses high reversibility. In contrast, the voltammograms of *Plasmodium*-infected erythrocytes showed remarkable peak splitting in both the cathodic and anodic regions, which was confirmed using square wave voltammetry as the detection mode (Fig. 4B). This voltammetry can be interpreted as the appearance of a second Fe(III)/Fe(II) couple (C_2_/A_2_) corresponding to Fe-heme species different from those in healthy specimens, which formed as a result of the *Plasmodium* infection. As judged by the value of the midpeak potential of the C_2_/A_2_ couple, this new species can be assigned to heme O^39^.

**Figure 5:**
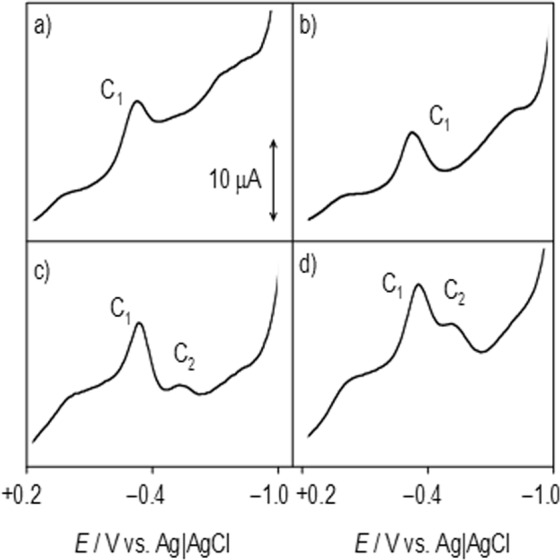
The square wave voltammograms of glassy carbon electrodes modified with **(a**) uninfected erythrocytes treated with INA at 2.0 µM, (**b**) uninfected erythrocytes treated with UNP at 20.0 µM, (**c**) *Plasmodium*-infected erythrocytes treated with INA at 2.0 µM, and (**d**) *Plasmodium*-infected erythrocytes treated with UNP at 20.0 µM, in contact with 0.1 M PBS at pH 7.4. The potential scan was initiated at +0.2 V in the negative direction; there was a potential step increment of 4 mV. The square wave amplitude was 25 mV with a frequency of 5 Hz.

The effect of INA and UNP on *Plasmodium*-infected erythrocytes is illustrated in Fig. 5, in which square wave voltammograms of glassy carbon electrodes modified with (a) uninfected erythrocytes plus INA, (b) uninfected erythrocytes plus UNP, (c) *Plasmodium*-infected erythrocytes plus INA, and (d) *Plasmodium*-infected erythrocytes plus UNP are shown. The relevant point to emphasize in these voltammograms is that the signal (C_2_) attributed to heme O in the samples of infected erythrocytes treated with INA and UNP is clearly lower than that in untreated *Plasmodium*-infected erythrocytes. These results suggest that INA and UNP interfere with the heme pathway, blocking the synthesis of heme O, and that this mechanism probably caused the observed decrease of parasitemia over 48 hours.

**Figure 6:**
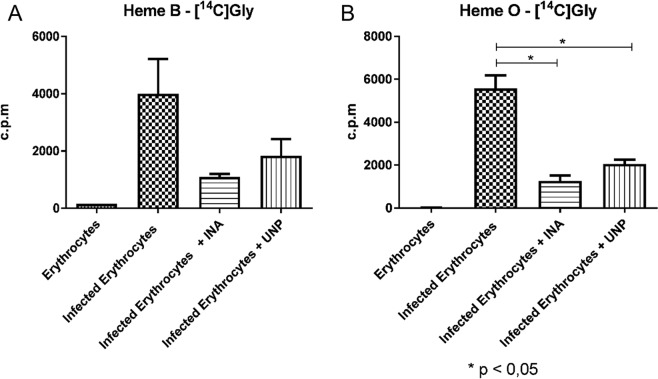
INA or UNP lead to the inhibition of heme B and heme O biosynthesis in parasites treated for 48 h. Cultures of *P. falciparum* were treated with 2.0 μM INA or 20 μM UNP and labeled with [U-^14^C]-glycine. The same amounts of treated and untreated parasites were applied to the column. The extract of the labeled schizonts was analyzed using C18 Vac columns and heme B was eluted with 80% of ACN while heme O was eluted with DMSO. **(A)** Heme B was detected in untreated parasites (Infected Erythrocytes) and in parasites treated with INA (Infected Erythrocytes + INA). The inhibition was approximately 75%. In parasites treated with UNP (Infected Erythrocytes + UNP), the inhibition was approximately 54%. No significant differences were observed in the inhibition of heme B. **(B)** heme O was detected in untreated parasites (Infected Erythrocytes). In parasites treated with INA (Infected Erythrocytes + INA), the inhibition was approximately 76% (p < 0.05), and in parasites treated with UNP (Infected Erythrocytes + UNP), the inhibition was approximately 64% (p < 0.05). The results presented correspond to three experiments. Erythrocytes: uninfected erythrocytes.

**Figure 7:**
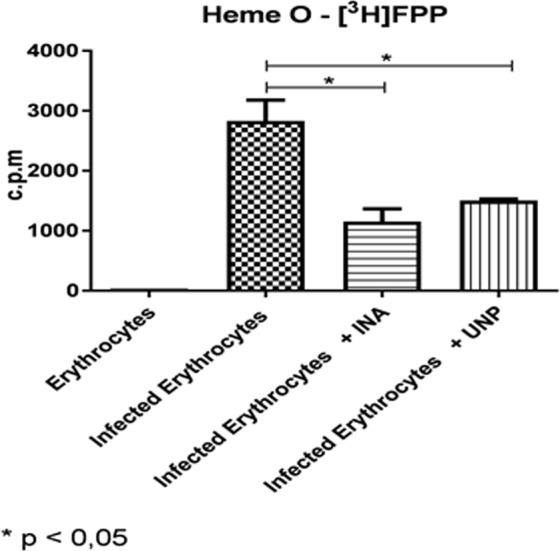
INA or UNP inhibit heme O biosynthesis in parasites treated for 48 h. Cultures of *P. falciparum* were treated with INA at 2.0 μM or 20 μM UNP and labeled with [^3^H]-FPP. The same amounts of treated and untreated parasites were applied to the column. The extract of labeled schizonts was analyzed with C18 Vac columns and heme O was eluted with DMSO. Heme O was detected in untreated parasites (Infected Erythrocytes). In parasites treated with INA (Infected Erythrocytes + INA), the inhibition was approximately 58% (p < 0.05), and in parasites treated with UNP (Infected Erythrocytes + UNP), the inhibition was approximately 52.5% (p < 0.05). The results correspond to three independent experiments. Erythrocytes: uninfected erythrocytes.

In view of the possible interference of UNP and INA with the heme group, as demonstrated by electrochemistry, we conducted a metabolic assay using schizonts labeled with [1-(n)-^3^H]-FPP or [U-^14^C]-glycine treated with INA or UNP at a concentration below the IC_50_values (2.0 μM INA or 20 μM UNP) (Figs. 6 and 7). This confirms that at the end of the treatment, a number of viable parasites remain, and partial inhibition of parasite growth still occurs. Then, each extract of the labeled schizonts was analyzed using C18 Vac columns. INA and UNP seemed to inhibit heme B synthesis (75% for INA and 54% for UNP, Fig. 6A). However, this effect was not statistically significant (p > 0.05) due to an uneven labelling of untreated parasites (see Supplementary Information Fig. S8). Since the parasites are able to acquire heme from the host, the effect of this treatment may not be a hindrance to the parasite^15,40^. Therefore, if the parasite’s heme biosynthesis is inhibited, the parasite could still recover heme from the host. Nevertheless, the biosynthesis of heme O in schizonts decreased significantly (Figs. 6B and 7), and the inhibition was 76% for INA and 64% for UNP when labeling with [U-^14^C]-glycine and 58% for INA and 52.5% for UNP when labeling with [1-(n)-^3^H]-FPP. This indicates that the inhibition mediated by INA and UNP are possibly not directly related to the inhibition of heme B or heme O synthesis but rather bind to heme accumulated in the food vacuole as previously also shown by Toyama and colleagues^17^.

The electrochemical analysis of heme O first showed the detection of the electrochemical signals corresponding to the farnesylated heme (heme O), specifically in the *Plasmodium*-infected samples. Interestingly, the potentials of the signals corresponding to heme O were higher than the redox potentials characteristic of the heme. This difference is due to the increase in stability contributed by the farnesyl group (present only in the heme O) when it is added to the heme group.

Second, the electrochemical analysis of the samples in the presence of UNP and INA (Fig. 5) and the comparison of the healthy and infected samples demonstrated that the presence of UNP and INA somehow interfered with the biosynthesis of heme O because its characteristic electrochemical signals mostly vanished. Importantly, the electrochemical behaviors of the cultures treated with UNP or INA are similar to the profile of the healthy sample, where only the signals corresponding to heme are observed (without the detection of heme O).

Concerning the apparent reduction in heme O synthesis, we tried to show an inhibition of this synthesis by adding UNP and INA in metabolic labeling assays (Figs. 6 and 7). The test showed a significant reduction in the parasite’s heme O production in the presence of drugs. Decreased formation of ALA (aminolevulinic acid) by addition of glycine does not appear to be the cause of death, since Nagaraj et. al., 2013^13^ used 3 μCi of [4-^14^C] ALA and this did not interfere with parasite growth.

The heme O-decreasing effect of INA and UNP treatment became visible by both labeling with [1-(n)-^3^H]-FPP or [U-^14^C]-glycine, and this indicates that farnesylation may be partially inhibited (Figs. 6B and 7). If INA and UNP inhibited the formation of heme O, it is possible that they also interfered with the mitochondrial potential. Therefore, we performed an experiment with the mitochondrial potential marker JC-1.

Parasites treated with INA and UNP for 48 hours apparently present a dose-dependent reduction in mitochondrial JC-1 incorporation. Therefore, the decrease in heme O may also be triggered by drug interference of mitochondrial function or by parasite death (Supplementary Information Fig. S9), suggesting that in this assay occurred non-specific parasite death rather than selective inhibition of heme O synthesis. The control culture treated with 15 nM chloroquine also showed a decrease of the mitochondrial function, in consequence of the parasite’s incapacity to polymerize heme and the concomitant production of reactive oxygen species^41^.

After the first 24 hours, UNP treatment caused a (dose dependent) decrease in mitochondrial function in relation to the control. Since UNP treatment is also related to the decrease of heme O, it may be hypothesized that the drug is acting in the mitochondrial potential. Notably, in the first 24 hours, no decrease in parasitemia was observed.

**Figure 8:**
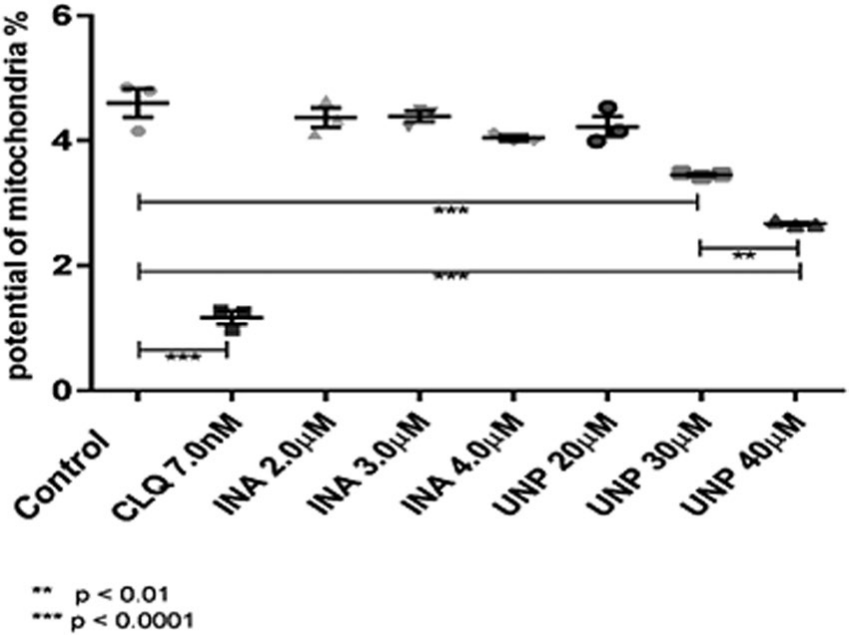
UNP interferes with the mitochondrial potential. Parasites treated with the indicated drug concentrations were incubated with the chromophore JC-1 and fluorescence was analyzed using a FACScalibur cytometer and the CellQuest Pro program, version 5.2. After a 24-hour incubation interval, we observed a significant difference in relation to the control. CLQ (chloroquine) was used at 7.0 nM and INA at 2, 3 and 4 μM. A significant difference between treatment with UNP at 30 and 40 μM, indicating dose dependence, was observed. Significance was assessed using Student’s t test. The results correspond to three independent experiments.

For INA treatment, there was also no appreciable effect after the first 24 hours. We cannot exclude that INA exerts an effect on the mitochondrial potential. Possibly, the dynamics of INA inhibition become visible only after more than 24 hours of treatment (Fig. 8).

Therefore, the probable inhibition of heme O by INA and UNP may in consequence affect the transport of electrons in the mitochondrial chain. As shown by Painter and colleagues, inhibition of mitochondria prevents the regeneration of ubiquinone in *P. falciparum* and this impaired regeneration of ubiquinone also necessary as the electron acceptor for dihydroorotate dehydrogenase, an essential enzyme for pyrimidine biosynthesis^42^.

In order to show that INA and UNP treatment also leads to decreased ubiquinone levels, a rescue experiment was performed where parasites were simultaneously treated with either INA or UNP and increasing concentrations of external decyl-ubiquinone. If INA and UNP at least partly inhibit parasite growth by decreasing internal ubiquinones, then addition of decyl ubiquinone should restore growth. This was in fact observed when 5 or 10 µM decyl- ubiquinone were added (Supplemental Fig. S10).

Heme conversion catalyzed by COX10 appears to be a limiting step in the rate of heme A formation^25^. Thus, the regulation of the abundance and activity of COX10 is likely to modulate the heme A biosynthesis pathway. It was established that the cofactor CoA2 acts together with COX10 in the hemylation of COX1. COX10 assembles into active oligomers in normal cells^26^, and the oligomerization of COX10 appears to be a key feature of the enzymatic activity of HOS. Thus, if heme O biosynthesis is at least partially inhibited by the drugs, the lower heme O levels are directly related to the low levels of heme A. This, in turn, may affect the mitochondrial potential, because the components of the complex IV are not biosynthesized and the parasite cannot obtain neither heme O nor heme A from the host.

## Conclusion

In this study, we demonstrated that *P. falciparum* has an active biosynthetic pathway for the production of heme O, the intermediate for heme A (unique cofactor of all known eukaryotic cytochrome c oxidases). Furthermore, we provided hints that INA and UNP are possibly responsible for the inhibition of heme B farnesylation besides other pleiotropic effects. In consequence, with less heme O, a decrease in parasitemia is observed. We propose that this pathway could be a valid target for novel drugs. The regulatory mechanisms that orchestrate heme metabolism of parasites remain poorly understood and are a frontier for future discovery.

## Methods

### *P. falciparum* culture

The *P. falciparum* clone 3D7 was cultured *in vitro* according to the method of Trager and Jensen^43^, except human serum was substituted by Albumax I (Invitrogen CA, USA) (0.5%)^44^. A gas mixture of 5% CO_2_, 5% O_2_, and 90% N_2_ was injected into bottles 75 cm^2^. The cultures (approximately 15% parasitemia) were initially synchronized in ring stages (1–10 h after invasion) by two treatments with 5% (w/v) D-sorbitol solution in water. The parasites were maintained in culture until the development of trophozoites (20–24 h after reinvasion) or schizonts (30–35 h after reinvasion) and then synchronized with Plasmagel^® ^^45^ (Laboratoire Roger Bellon, Neuilly sur Seine, France).

### Metabolic labeling

Synchronous cultures of *P. falciparum* were labeled with [1-(n)-^3^H]-geranylgeranyl pyrophosphate ([1-(n)-^3^H]-GGPP) (3.125 μCi/mL, 16.5 Ci/mmol, Amersham Bioscience, UK) or [1-(n)-^3^H]-farnesyl pyrophosphate ([1-(n)-^3^H]-FPP) (3.125 μCi/mL, 14.0 Ci/mmol, Amersham Bioscience, UK) or L-[^14^C(U)]-glycine (112.7 μCi/mL, 112.7 mCi/mmol, Amersham Bioscience, UK) in normal RPMI 1640 medium in trophozoite stages (20% parasitemia) for 10–14 h and were then recovered as schizonts. Subsequently, the parasite cultures were centrifuged at 800 × *g* for 10 min at 22 °C. The intraerythrocytic stages were purified using magnetic columns^46^ and submitted to different extraction methods of gibberellin, heme B or heme O, which are further described below. As a control, the same procedure was carried out with uninfected erythrocytes. The herein used erythrocytes were older than 15 days. Therefore, no reticulocytes which may contain trace amounts of heme synthesis could be detected.

### Extraction of gibberellin

For the extraction of gibberellin, schizont stages labeled with [1-(n)-^3^H]-GGPP as described above were purified using magnetic columns and homogenized with 1.0 M Tris-HCl, pH 7.2, containing 2% Triton X-100 and incubated for one hour at 4 °C. After centrifugation (25 min at 8.000 × *g* and 4 °C), the supernatant was adjusted to pH 2.5. The separation procedure between the remaining aqueous extract and the diethyl-ether phase was conducted using the method described by Barthe and Bulard,^47^, and this fraction was called PfexG.

### RP-HPLC

The separation of gibberellin by gradient RP-HPLC was adapted from the isocratic system described by Bhalla and Singh^48^, where the solvent was initially 90% acetonitrile (ACN) and 10% H_2_O at a flow of 0.5 mL/min for 45 min at 30 °C over a YMC C18 ODS-A column (5 µm, 300 Å, 3.0 mm × 150 mm). The wavelength used for analysis was 206 nm, and commercial gibberellin 3 (GA3) and 4 (GA4) (Sigma-Aldrich, Darmstadt, Germany) was used as a standard.

PfexG was co-injected with standard GA4, and the components were chromatographically separated by RP-HPLC. The samples were dried at 50 °C, resuspended in 0.5 mL of scintillation liquid (BetaplateScint, Perkin Elmer, Groningen, Netherlands) and analyzed in a Beckman^®^ scintillator unit.

### *In silico* analysis

The genome of *P. falciparum* was obtained from *Plasmodium* Genomic Resource DataBase (http://plasmodb.org/plasmo/). Sequences from the ‘TriTryps’ genome projects were obtained from GeneDB (http://www.genedb.org/) and TriTrypDB (http://tritrypdb.org/tritrypdb/)^49^. The *S. cerevisiae* sequence *ScCOX10* (YPL172C) was obtained from the *Saccharomyces* Genome Database (www.yeastgenome.org). The *E. coli* sequence (NC_000913.3), *B.subtilis* sequence (NC_000964.3), *A. thaliana* sequence (NM_130015.3) and *Homo sapiens* sequence (NM_001303.3) were obtained from NCBI (http://www.ncbi.nlm.nih.gov). Other sequences were obtained from The Universal Protein Resource (UniProt - http://www.uniprot.org): A0A1D3SEW6 - *P. berghei*, A0A0J9STT8 – *P. vivax* Brazil I, W7JZG5 – *P. falciparum* (isolate NF54), W7AUT1 – *P. vinckeipetteri*, A0A1D3U8S5 – *P. ovale*, V5BB79 – *T. cruzi*, Q4QAY6 – *L. major*, A4HD36 – *L. braziliensis*, G0VC94- *N. castellii*, A5E2Y6 – *L. elongisporus*, A0A061D8D2 - *B. bigemina* and Q4UA41 – *T. annulata*.

For multiple amino acid sequence alignments, ClustalW 2.0.12 software was used^50^. The similarity and identity percentages were calculated using Ident and Sim software (http://www.bioinformatics.org/sms2/ident_sim.html).

The transmembrane domain predictions for the COX10 enzymes were generated using software for topology prediction, TMHMM 2.0 (http://www.cbs.dtu.dk/services/TMHMM-2.0/).

Phylogenetic and molecular evolutionary analyses were conducted using MEGA6 software^51^.

### Immunofluorescence and localization of GFP-tagged COX10

For immunofluorescence analysis, parasites were fixed with 4% EM grade paraformaldehyde and 0.0075% EM grade glutaraldehyde in PBS for 30 min as described by Tonkin *et al*.^30^. Parasites were then washed with PBS and blocked with 3% BSA for 1 h. Afterwards, cells were incubated with the anti-apicoplast primary antibody (dilution 1:1000, formulated according to Tonkin *et al*.^30^, and developed by FastaBio Ltda, Ribeirão Preto, Brazil) diluted in 0.1% BSA, 0.001% saponin in PBS for 1 hour at 24 °C. Cells were then washed three time with PBS and incubated with the secondary antibody Alexa Fluor® 488 and DAPI 2 μg/ml also diluted in 0.1% BSA and 0.001% saponin in PBS for 45 min at 24 °C. After three washes with PBS the slides were mounted and sealed. The images were acquired in a fluorescence microscope Axio observer Z1 and processed using the Photoshop version 5.

For mitochondria localization parasites were incubated with Mitotracker, (Molecular Probes, Oregon, USA - dilution 1:10,000) for 30 min at 37 °C. Afterwards, parasites were incubated with DAPI final concentration of 2 µg/mL at 37 °C for 1 h. Finally, parasites were washed 3 times with PBS/saponin. Images were also acquired in a fluorescence microscope Axio observer Z1 and processed using the Photoshop version 5.

### Vector construction

To obtain the transfection vector containing the desired cox10 gene of *P. falciparum*, the cox10 ORF was PCR-amplified (primers 5′-agatctATGGGATTTAATAAGATTTTTCC and 5′-ctgcagcTTTAAATGTTCTTTTTATCAAATGTAGG, program 94 °C, 40 s, 54 °C, 40 s, 65 °C 1 min 30 s, 30 cycles) on genomic DNA from PF3d7, using Elongase enzyme mix (Thermo Scientific/Invitrogen, Carlsbad, CA, USA) and cloned into pGEM® T-easy (Promega, Wisconsin, USA). The fragment was sequenced and a fragment containing the correct sequence was excised using via the introduced *Bgl*II and *Pst*I sites and ligated in the pRESA-GFP/HA vector^52^ and cut with the same enzymes.

The construct was transfected into *P. falciparum* 3D7 as described^53^. Integrated parasites were checked for green fluorescence. Given that the 5′ end or the promoter region of the cox10 ORF was not modified, the fused protein COX10-GFP-HA is supposed to be expressed at levels similar to the unmodified locus^54^. The vector construction is shown in Supplementary Information Fig. S1. Note that in this construct, only parasites with recombinant loci show green fluorescence, since there is no promoter included in the 5′ position of the COX10-GFP-HA fusion gene.

### Heme B and heme O Standard Preparations

Heme B and heme O are not commercially accessible. The expression vector pET3a containing the CtaB sequence of *Bacillus subtilis* was used to transform *E. coli* BL21 cells in order to prepare heme O. Heme B and heme O were extracted from transformed bacteria with acetone and hydrochloric acid and then separated on Sep-Pak Vac C18 columns (Waters Corporation, Milford, Massachusetts USA), which is further described below. The purity of the fractions eluted by 80% ACN for heme B and DMSO for heme O from the bacterial extract was assessed by mass spectrometry, as described below. Standardized methodology using BL21 bacteria transformed with pet9a/ctb plasmid provided by John Wright and Eric L. Hegg of the Laboratory of Biochemistry and Molecular Biology at Michigan State University, United States (Supplementary Information Figs. S5–S7).

### Extraction of heme B and heme O

For the extraction of heme B and O, schizont stage parasites labeled with [1-(n)-^3^H]-FPP or [U^14^C]-glycine and purified by magnetic columns were lysed with 0.1% saponin and resuspended in 0.2 M Tris-HCl pH 8.0 with 10% sucrose. Bacterial lysates containing heme O and heme B were treated equally. Then, a mixture of acetone/HCl (95:5) was added in a proportion of 40% of the lysate/60% acetone/HCl, and the solution was kept on ice for 90 min. Then, the parasite or bacterial lysate was centrifuged at 8,000 × *g* for 10 min at 4 °C to extract heme B and heme O. Uninfected erythrocytes labeled with the same radioactive tracers and processed under equal conditions were used as a control. In total, 1 × 10^9^ schizont stage parasites and the same quantity of uninfected erythrocytes were analyzed with mass spectrometry in the same way.

### Separation of heme B and heme O

The separation of both types of heme was performed using C18 Vac columns. The column was pre-washed with 2 mL of 25% ACN. The supernatant from the heme extraction was collected and passed through this column. Then, the column was washed with 5 mL of 25% ACN. Then, 80% ACN was used to elute heme B, and DMSO was used to elute heme O^33^. For bacteria, the volume of solvents used was three times higher.

### Mass spectrometry

#### LC-MS/MS

Heme samples were analyzed using a LC-MS/MS system of an ACCELA 600 quaternary pump LC connected to a triple quadrupole mass spectrometer, the TSQ Quantum Max (Thermo Scientific, Bremen, Germany) via an ESI ionization source.

The separation was performed using a Hypersil Gold C18 Column (100 × 2.1 mm, i.d., 5 μm; Thermo Scientific, Bremen, Germany). The column temperature was kept at 25 °C. The injection volume was 5 μL.

The mobile phase used to separate the compounds contained water with 0.02% of acetic acid (A) and ACN with 0.02% of acetic acid (B). The composition of solvent B varied as follows: 0–2 min, held at 10%; 2–20 min, from 10 to 90% linear gradient; and 20–30 min, held at 10%. The flow rate was 0.2 mL/min.

The TSQ Quantum Max mass spectrometer was operated in product ion MS scan mode with positive polarity. The MS operating parameters were a spray voltage of 3500 V, a vaporizer temperature of 375 °C, a sheath gas pressure of 60 (arbitrary units), an ion sweep gas pressure of 2.0 (arbitrary units), an auxiliary gas pressure of 55 (arbitrary units), a capillary temperature of 224 °C, and a capillary offset of 35 V.

#### MALDI-TOF/TOF

The samples were analyzed using a MALDI-TOF/TOF Autoflex speed smartbeam mass spectrometer (Bruker Daltonics, Bremen, Germany) with the FlexControl software (version 3.3, Bruker Daltonics). Spectra were recorded in positive reflector mode (laser frequency, 500 Hz; extraction delay time, 130 ns; ion source 1 voltage, 19.0 kV; ion source 2 voltage, 16.8 kV; lens voltage, 7.9 kV; reflector 1 21.0 kV; reflector 2 9.35 kV; and mass range, 500 to 2500 Da). For each spectrum, 5000 shots in 500-shot steps were summed from different positions of the target, collected and analyzed. All spectra were calibrated using a Peptide Calibration Standard (Bradykinin [M + H] + = 757.3991, Angiotensin II [M + H] + = 1046.5418, Angiotensin I [M + H] + = 1296.6848, Substance P [M + H] + = 1347.7354, Bombesin [M + H] + = 1619.8223, Renin Substrate [M + H] + = 1758.9326, ACTH clip 1–17 [M + H] + = 2093.0862, and ACTH clip 18–39 [M + H] + = 2465.1983) (Bruker Daltonics, Bremen, Germany).

For MS/MS experiments, the instrument was set to LIFT mode (laser frequency, 200 Hz; extraction delay time, 120 ns; ion source 1 voltage, 6.0 kV; ion source 2 voltage, 5.3 kV; lens voltage, 3.0 kV; reflector 1, 27.0 kV; reflector 2, 11.6 kV; lift 1, 19.0 kV and lift 2, 4.40 kV), and 2000 shots were summed in 500-shot steps for each acquired spectrum.

### Electrochemical study

Electrochemical measurements were performed at 298 ± 1 K in a thermostatic cell with CH 660I equipment. A BAS MF2012 glassy carbon working electrode (GCE) (geometrical area 0.071 cm^2^), a platinum wire auxiliary electrode and an Ag/AgCl (3 M NaCl) reference electrode were used in a conventional three-electrode arrangement. Cyclic and square wave voltammetry (CV and SWV, respectively) were used as detection modes. Eventually, semi-derivative convolution of the data was performed in order to increase peak resolution.

Thin films of lyophilized erythrocytes and parasitized erythrocytes treated with 2.0 µM INA (Sigma-Aldrich, St. Louis, MO) or 20 µM UNP (Sigma-Aldrich, St. Louis, MO) or untreated were prepared on glassy carbon electrodes following a previously reported procedure^55^. Essentially, 20 µL of the dispersed erythrocyte material (1 mg/mL) in ethanol were applied, and the solvent was allowed to evaporate. As a result, a uniform, fine coating adhered to the basal electrode. Aqueous 0.10 M potassium phosphate-buffered saline (PBS) at a physiological pH of 7.2 that was previously degassed by bubbling with argon for 10 min was used as a supporting electrolyte.

### Inhibition tests with INA and UNP

Toyama *et al*.^17^ determined the IC_50_ values for INA and UNP in *P. falciparum*. Three concentrations of each plus a lower and a higher concentration as well as a concentration at the IC_50_ value, were evaluated and confirmed in our laboratory, and two controls, one without the drug and another with the vehicle, were used.

Another test was conducted in order to evaluate whether the inhibition of parasite growth caused by INA and UNP was reversible. We also assessed whether the parasite recovered by acquiring exogenous gibberellin contained in the medium upon INA or UNP treatment. For this, different concentrations (10 µM–150 µM) of gibberellin (G4) were added to cultures. These experiments were carried out in triplicate in independent biological triplicates. After the confirmation of the IC_50_ values, the parasite culture was treated with the drugs (INA at 2.0 µM and UNP at 20 µM) for a total of 48 h and labeled with radioactive precursor [1-(n)-^3^H]-FPP or [U-^14^C]-glycine for the last 12 h in the presence of INA and UNP. The biosynthesis of heme B and heme O were analyzed using C18 Vac columns. The same amounts of treated and untreated parasites were applied to the column.

The resulting samples were processed as described above, air-dried at 50 °C, resuspended in 0.5 mL of scintillation liquid (Betaplate Scint, Perkin Elmer, Finland) and analyzed using a Beckman® scintillator unit.

### Parasite growth recovery with external decyl-ubiquinone under INA and UNP treatment

Specificity of INA and UNP action was shown by addition of decyl-ubiquinone. For this, cultures were treated for 48 h with 3 µM INA or 30 µM UNP and concomitantly with 1, 5 or 10 µM decyl-ubiquinone. Parasite growth was monitored by Giemsa stained blood smears after 48 h.

### Mitochondrial potential

Initially, *P. falciparum* was treated with INA (2.5 μM and 5.0 μM), UNP (25 μM and 50 μM) and chloroquine (CLQ) (5 nM and 15 nM) (Sigma-Aldrich, St. Louis, MO), and samples were collected and centrifuged after 48 hours of treatment. Infected erythrocytes with the initial parasitemia of 25% were incubated with 2 μM of JC-1 (Sigma-Aldrich) for 30 minutes at 37 °C with stirring every 10 minutes. Afterwards, the cells were washed 4 times with PBS. After the fourth wash, part of the sample was incubated with 10 μL/mL of Hoechst stain (10 mg/mL) for 60 seconds and washed a fifth time. Samples incubated with JC-1 and Hoechst were observed by flow cytometry to verify whether the action of the drug is related to the mitochondrial potential^56^.

In addition, the same test was performed with INA (3.0 μM and 4.0 μM), UNP (30 μM and 40 μM) and CLQ (15 nM) to verify if the mitochondrial potential was also affected during the first 24 hours of treatment using the flow cytometer Guava EasyCyte Plus.

### Statistical analysis

For variables with a normal distribution, Student’s t-test was applied to compare means between untreated infected erythrocytes and INA or UNP treated infected erythrocytes. Statistical analyses were completed using the GraphPad PRISM^®^ 5.3 software.

## Supplementary information

Supplementary info
