# Supplementary material for: Biosynthesis of heme O in intraerythrocytic stages of Plasmodium falciparum and potential inhibitors of this pathway
Source: Sci Rep. 2019 Dec 17;9:19261. doi: 10.1038/s41598-019-55506-y (PMC6917786; doi:10.1038/s41598-019-55506-y)
Supplement: Supplementary file 1 — Supplementary info [file 41598_2019_55506_MOESM1_ESM.doc]

Supplementary data

**Biosynthesis of heme O in intraerythrocytic stages of *Plasmodium falciparum* and potential inhibitors of this pathway**

Raquel M. Simão-Gurge^1^; Gerhard Wunderlich^1^; Julia A. Cricco^2^; Eliana F. Galindo Cubillos^1^, Antonio Doménech-Carbó^3^; Gerardo Cebrián-Torrejón^3,4^; Fernando G. Almeida^1^; Brenda A. Cirulli^2^; Alejandro M. Katzin^1#^

^1^Department of Parasitology, Institute of Biomedical Sciences, University of São Paulo, Brazil. Instituto de Biología Molecular y Celular de Rosario (IBR), Consejo Nacional de Investigaciones Científicas y Técnicas CONICET – Facultad de Ciencias Bioquímicas y Farmacéuticas, Universidad Nacional de Rosario. Argentina. ^3^Departament of Analytical Chemistry, Faculty of Chemistry, University of Valencia, Spain. ^4^Laboratoire COVACHIM-M2E EA 3592, Université des Antilles, 97157 Pointe-à-Pitre Cedex (Guadeloupe), France.

^#^ Corresponding author: Alejandro Miguel Katzin.

^#^To whom correspondence should be addressed: amkatzin@icb.usp.br

^#^Corresponding author’s address: Department of Parasitology, Institute of Biomedical Sciences, University of São Paulo, Av. Lineu Prestes 1374, CEP 05508-000, São Paulo, SP, Brazil. Fax: +55 11 3091 7417.


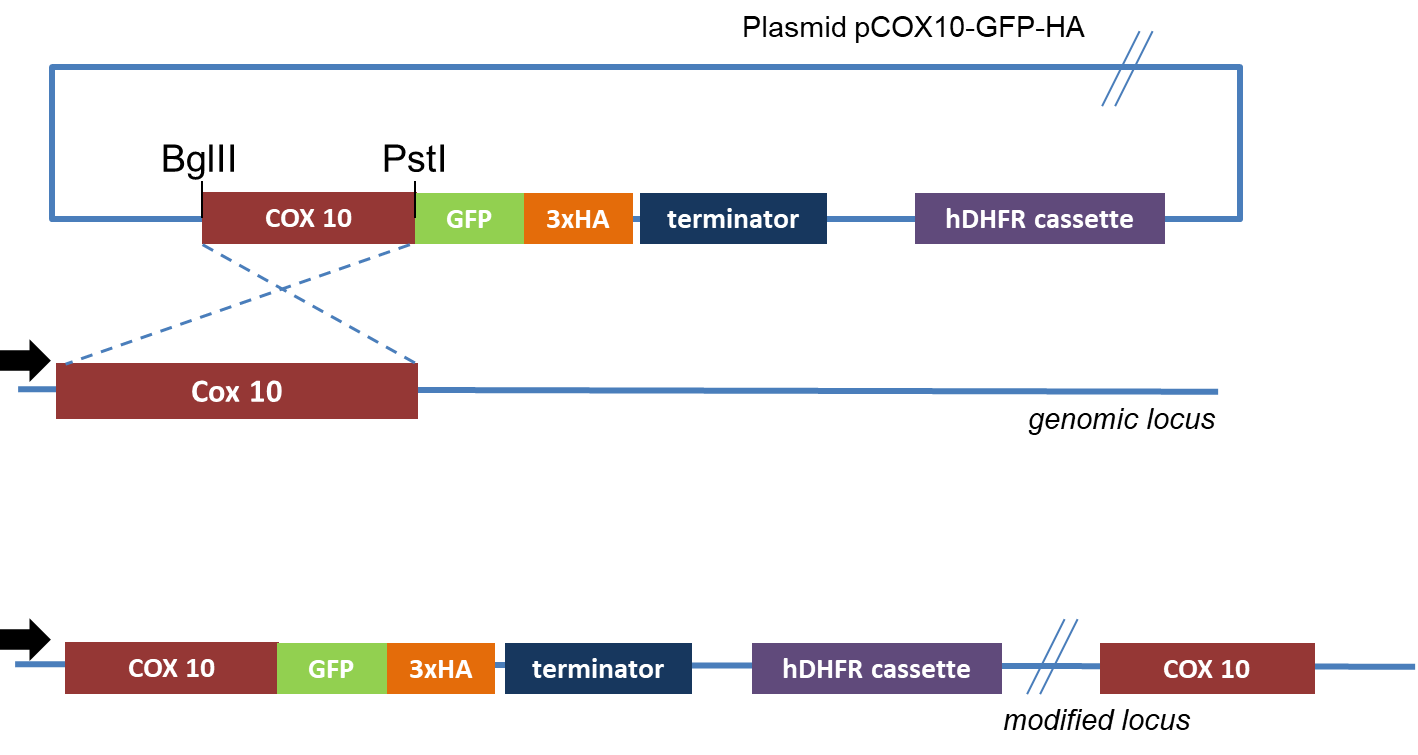


Figure S1: Scheme of the recombination in the locus COX10. Above, the plasmid used for single crossover recombination and the probable insertion of the plasmid in the original locus. Below, suggested structure of the modified locus. Note that the original 5’ upstream sequence (black arrow) is maintained and that the parasite only emits green fluorescence if the plasmid recombines in the locus since the plasmid itself has no functional promoter for COX10.


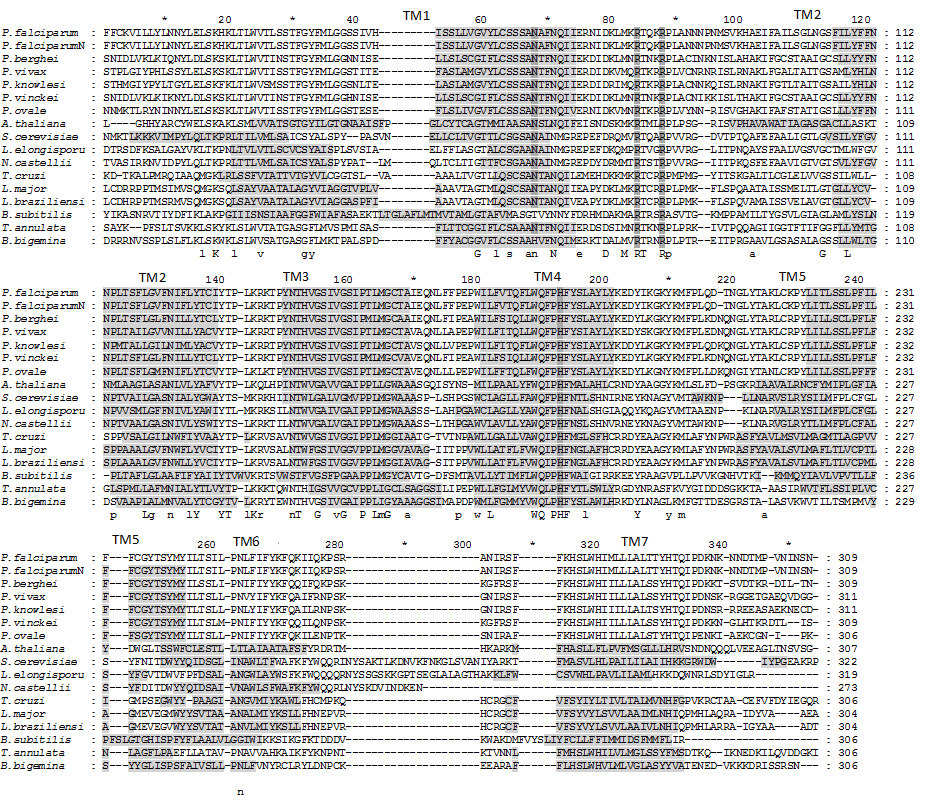


**Figure S2: The alignment of COX10 sequences from different organisms.** The sequences from *Plasmodium* and *Leishmania* species, *A. thaliana, S. cerevisiae*, *L. elongisporus, N. castellii, T. cruzi*, *B. subitilis*, *T. annulata*and *B. bigemina* show conserved areas; these include the transmembrane regions (TM). Most of COX10 proteins present 7 TM regions or *P. falciparum* COX10 presents 5 TM regions. The conserved residues that are involved in the function of COX10 proteins are shown in the darkest gray, that correspond to N196, R212, R216 and H317 residues in *S. cerevisiae* COX10.


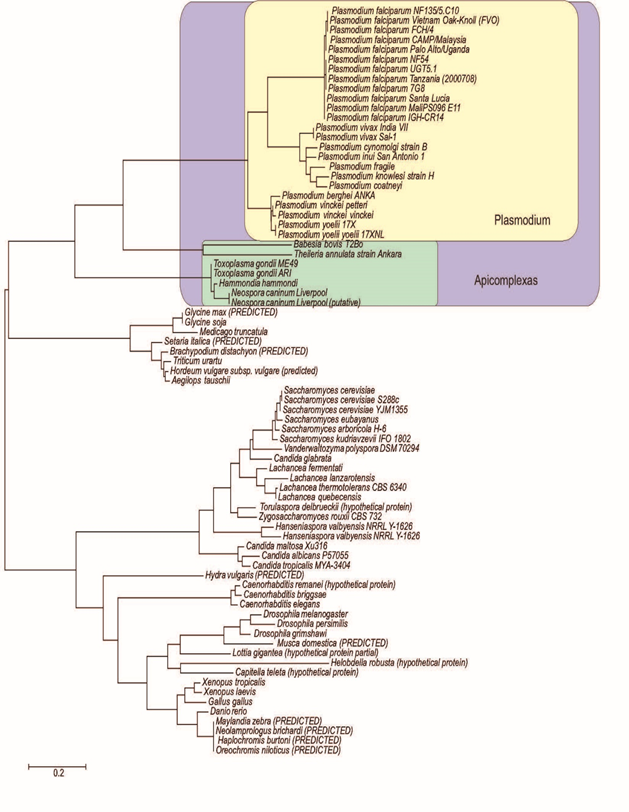


**Figure S3: Phylogenetic tree of Cox10 sequences.** The tree shows the evolutionary relationship among the cox10 sequences of selected different species from different phyla.

A


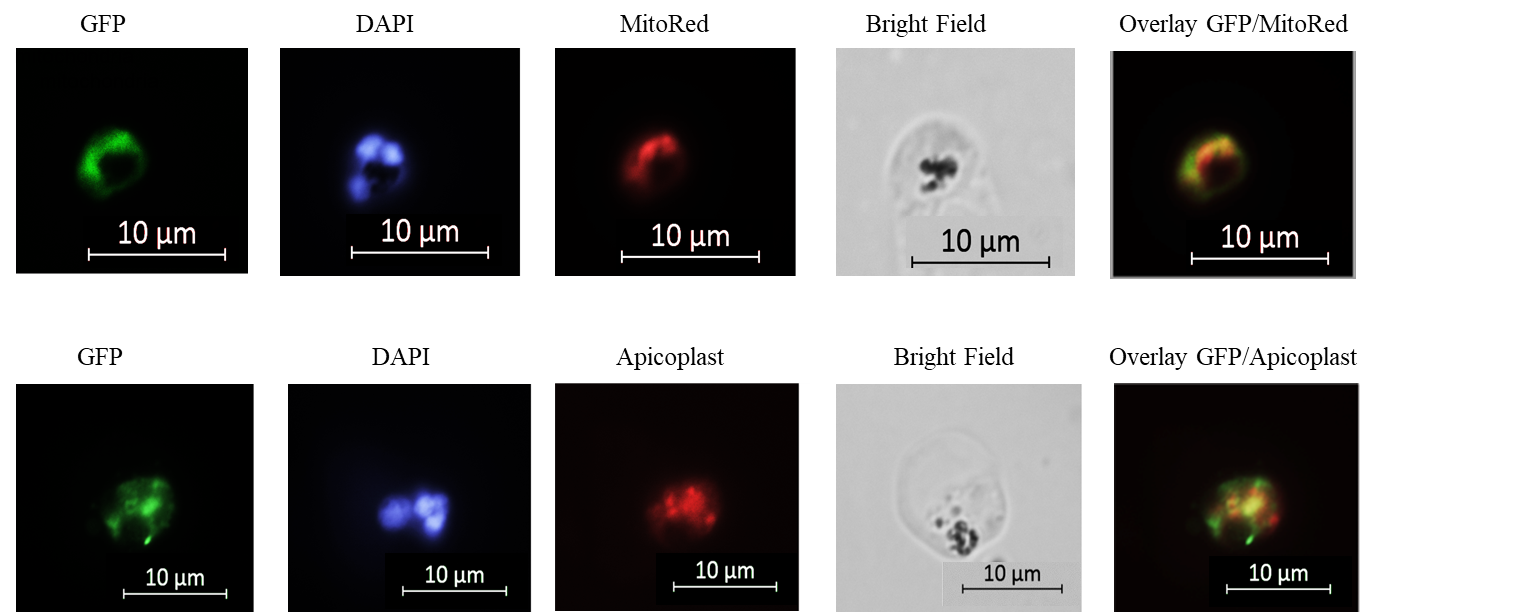


B


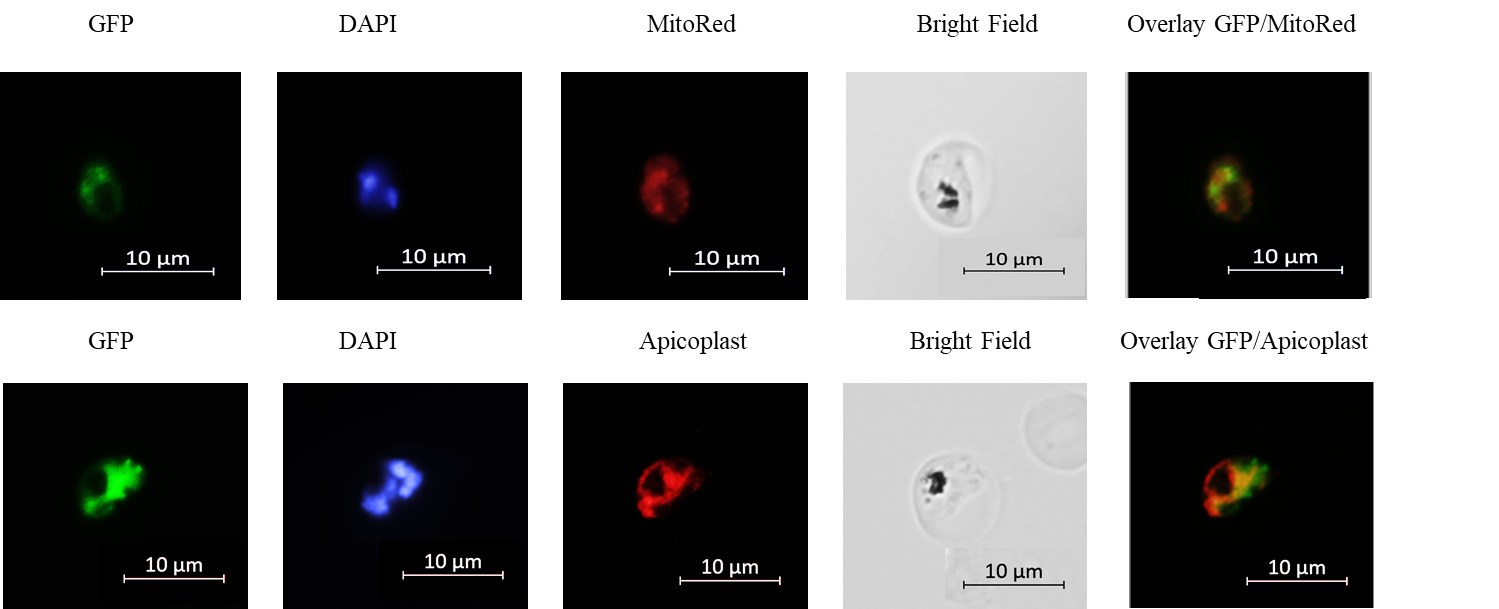


**Figure S4 – The localization of GFP-tagged COX10. Parasites labeled with DAPI and MitoRed (upper panels A and B) or with an apicoplast-specific antibody (bottom panels, A and B) formulated according to Tonkin et al.,^30^.** A and B represent exposures of different parasites. The COX10 images show a larger area of GFP overlap with the mitochondria (MitoRed, upper panels A and B), a small overlapping area with the nucleus (DAPI) and an overlap with the apicoplast that exceeds this organelle’s limits, indicating that the enzyme is in a larger organelle (anti-apicoplast, bottom panels A and B). Note that in dividing cells, Acyl carrier protein (ACP)-carrying apicoplasts are seen as distinct spots which are not directly coincident with the COX10 signal (green), suggesting a differential localization of PfCOX10 and ACP in apicoplasts. Pictures were taken with a Zeiss LSM-780 NLO Multifoot confocal microscope. The overlay is the combined signal of GFP and MitoRed (upper panels), or the anti-apicoplast and GFP (bottom panels).


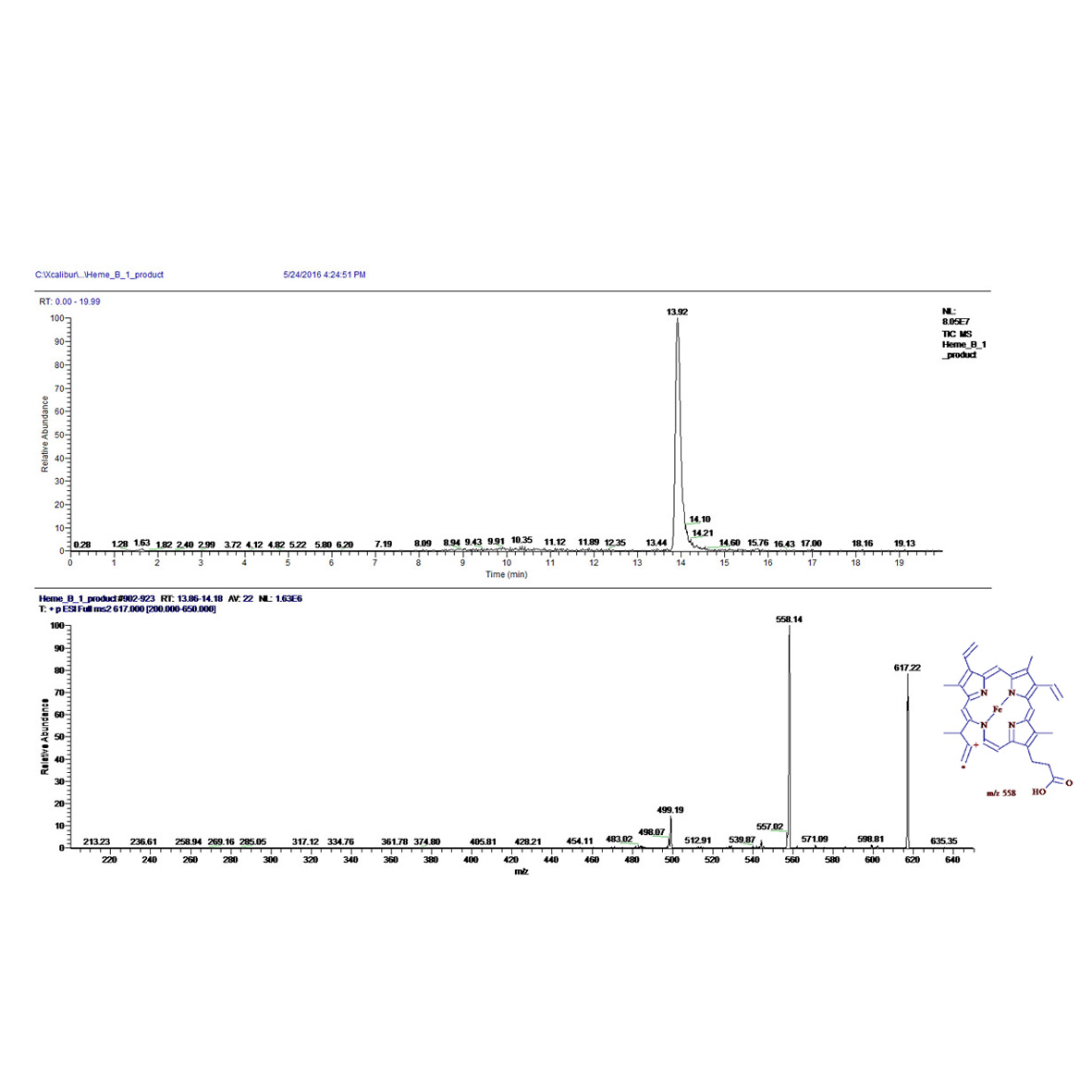


**Figure S5: LC-MS/MS analysis of bacterial extracts**. Material was loaded on C18 Vac columns and eluted with 80% ACN (heme B). Analysis of this fraction by LC-MS/MS and revealed its retention time at 13.92 min (arrow, 617.22, compatible with its calculated mass).


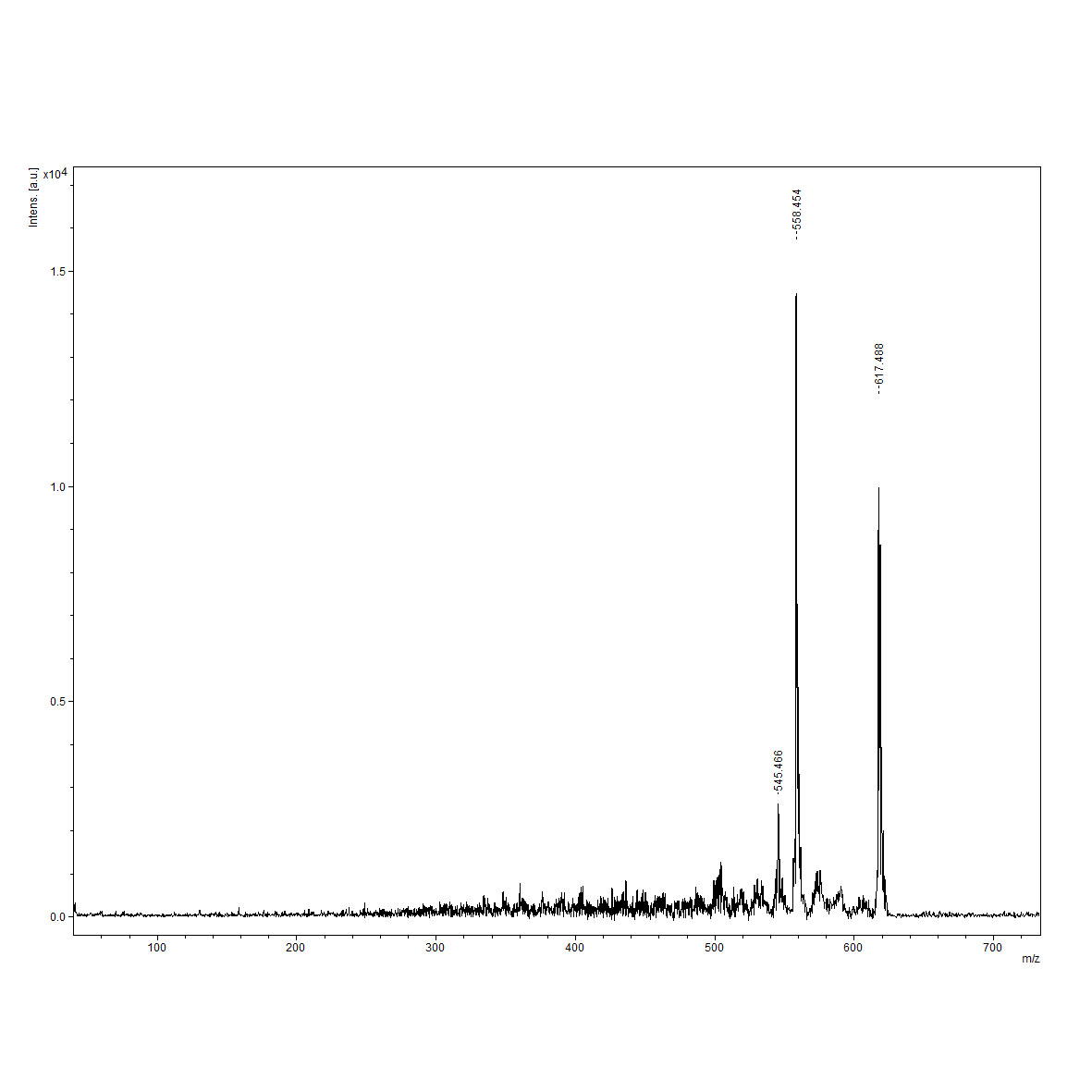


**Figure S6: MALDI-TOF/TOF analysis of bacterial extract**. Bacterial extracts were loaded on C18 Vac column and eluted with 80% ACN (heme B). Analysis of this fraction by MALDI-TOF/TOF showed a peak with a mass of 617, compatible with heme B.


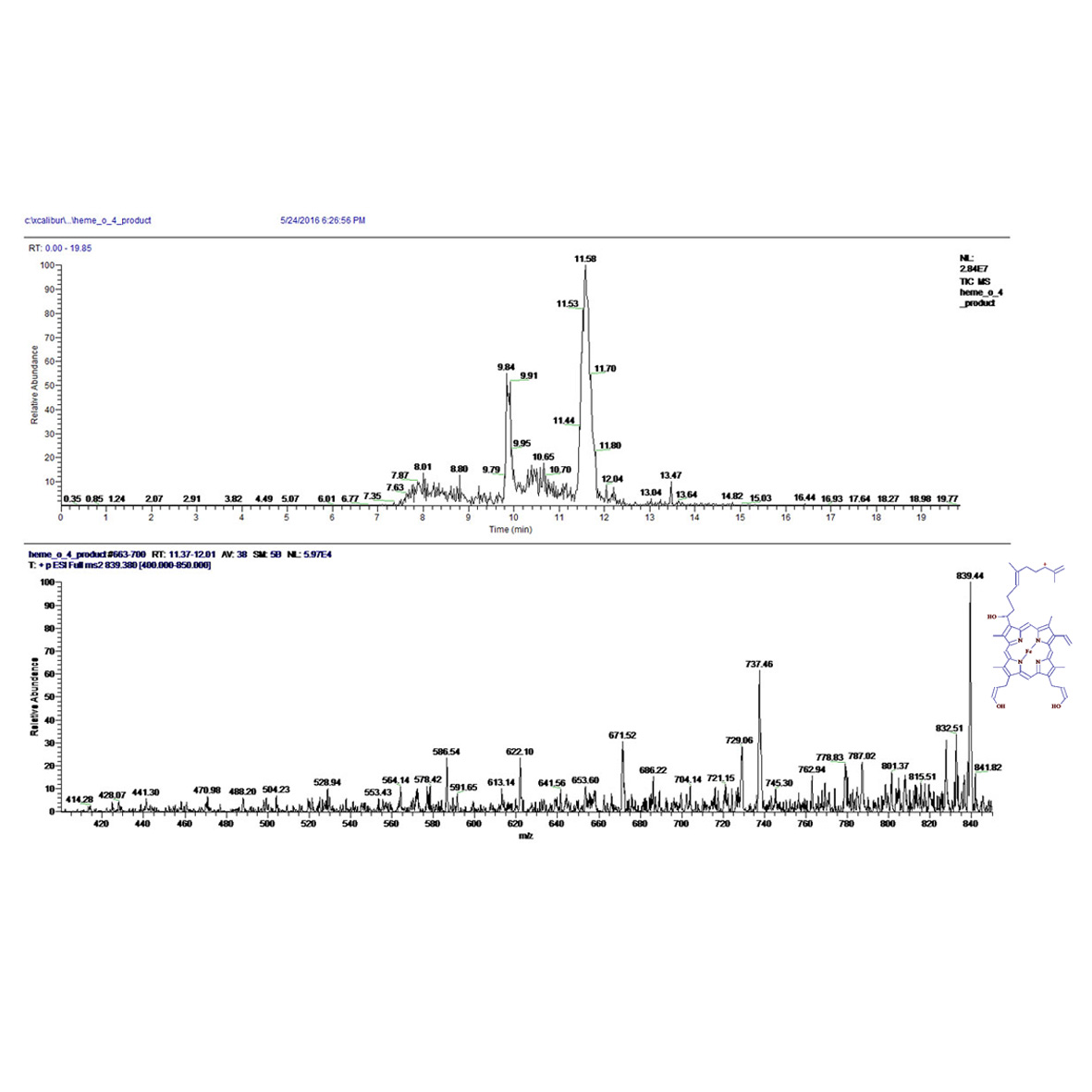


**Figure S7: LC-MS/MS analysis of bacterial extracts**. Here, material was loaded on C18 Vac columns and heme O was eluted with DMSO. The fraction was analyzed by LC-MS/MS and showed the m/z of heme O and its retention time of 11.58 min (peak, 839.44, compatible with its calculated mass).


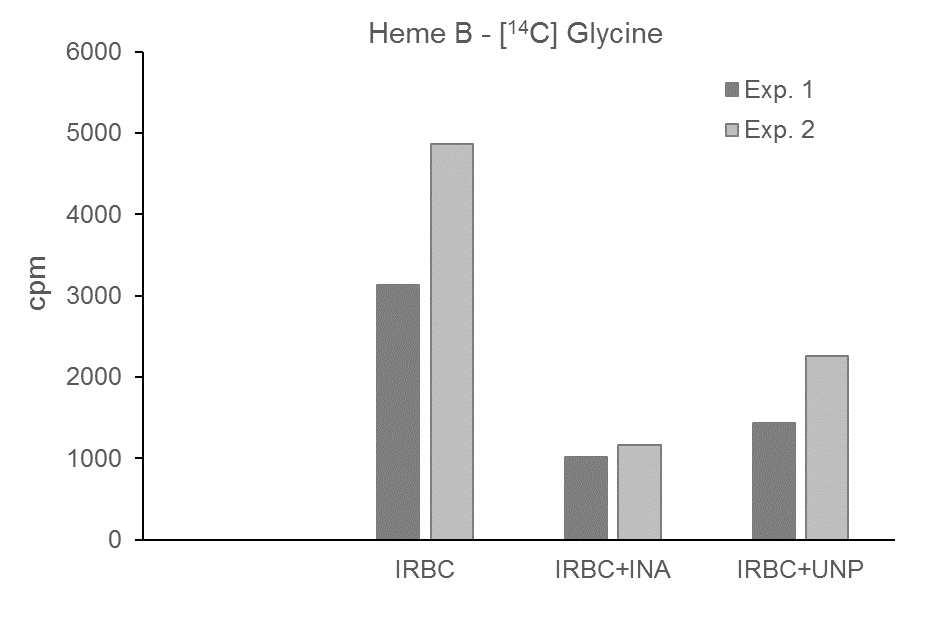


**Figure S8–INA or UNP lead to the inhibition of heme B biosynthesis in parasites treated for 48h.** Cultures of *P. falciparum* were treated with 2.0 μM INA or 20 μM UNP and labeled with [U-^14^C]-glycine. The same amounts of treated and untreated parasites were applied to the column. The extract of the labeled schizonts was analyzed using C18 Vac columns and heme B was eluted with 80% of ACN. Heme B was detected in untreated parasites (**IRBC**) and in parasites treated with INA (**IRBC+INA**) the inhibition was approximately 68% and 76% respectively. In parasites treated with UNP (**IRBC+UNP**), the inhibition was approximately 55% and 54% respectively. Data from two independent experiments are shown.


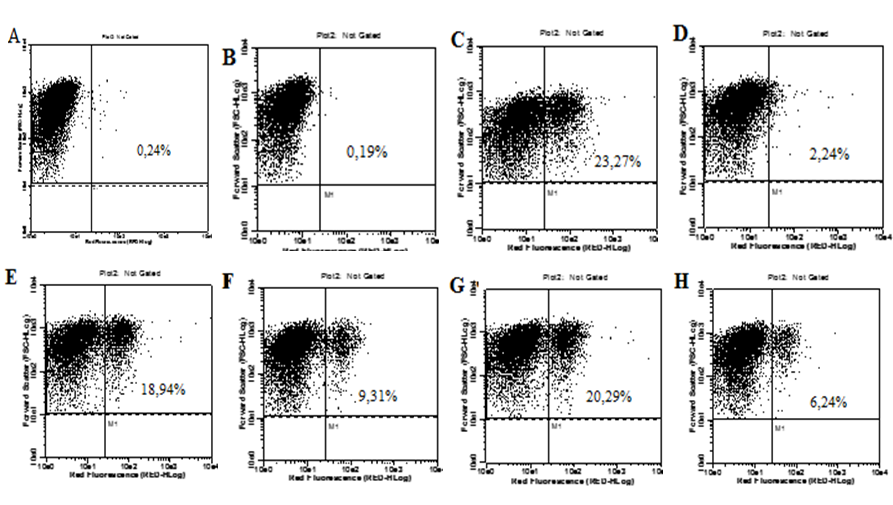


**Figure S9: Treatment of parasites for 48 h decreases their mitochondrial potential.** In the upper right quadrant of each graph: parasitized red cells, and the upper left quadrant: non-parasitized red blood cells. (A) Non-parasitized erythrocytes. (B) Non-parasitized erythrocytes without JC-1; (C) Parasitized erythrocytes with JC-1; (D) Parasitized erythrocytes with JC-1 and 15 nM CLQ; (E) Parasitized erythrocytes with JC-1 and INA 2.0 μM; (F) Parasitized erythrocytes with JC-1 and INA at 5.0 μM; (G) Parasitized erythrocytes with JC-1 and 25 μM UNP; (H) Parasitized erythrocytes with JC-1 and 50 μM UNP. This experiment showed a dose-dependent relationship for decreased parasitemia and mitochondrial potential, since at lower drug concentrations, the decrease in parasitemia and mitochondrial potential was lower than that detected at higher drug concentration.

**Figure S10**: **Externally added decyl-ubiquinone restores growth in UNP/INA treated parasites.** Synchronized parasites were treated with 3 µM INA or 30 µM UNP as described and were added or not with 1, 5 or 10 µM decyl-ubiquinone. The graph represents the results of two experiments.
